# Impact of green factors on undergraduate students’ green behavioral intentions: A hybrid two-stage modeling approach

**DOI:** 10.1016/j.heliyon.2023.e20630

**Published:** 2023-10-05

**Authors:** Sanjoy Kumar Roy

**Affiliations:** General Education Department, City University, Khagan, Birulia, Savar, Dhaka-1216, Bangladesh

**Keywords:** Green consumer, Green product, Green behavioral intention, Green environmental awareness, TPB, NCA

## Abstract

Consumers' unsustainable behavior contributes to environmental degradation and impedes sustainability. Using green products is one way to reduce this effect and promote environmental growth. Therefore, this study aims to investigate the green factors that impact young customers' behavioral intentions regarding green products. For this purpose, the researcher designed a research model based on the expanded theory of planned behavior (TPB). The study adopted a two-stage, hybrid model using partial least square-structural equation modeling (PLS-SEM) and necessary condition analysis (NCA) to evaluate and validate the results. A sample of 382 undergraduate students was gathered using a convenience sampling approach. The results reveal that green TPB perception (GTP), green price sensitivity (GPS), green product trust (GPT), and green product value (GPV) are significantly and positively correlated with green behavioral intention (GBI). This study's main contribution is adding a brand-new higher-order construct, GTP, in the environmental and marketing literature and validating its effect on GBI. Again, environmental awareness moderates the association between GPS and GBI. Furthermore, the NCA's findings indicated that these variables are necessary to various degrees for students' GBI. Decision-makers may use the study's insights to create successful regulations to better understand young customers and develop appropriate green initiatives for sustainable development.

## 1 Introduction

The current world is facing the problem of environmental issues [1]. Due to human activities, society is seriously threatened [2]. Environmental issues are made worse by human activity, which also affects the growth of plants and animals [1]. Environmental problems such as eutrophication, acid rain, water and air pollution, ozone depletion, declining flora and fauna, and global warming have grown in importance [3]. For example, the greenhouse effect, produced by people directly or indirectly, is responsible for the ongoing increase in world temperature. Again, overpopulation has put a tremendous strain on ecological systems [4]. Therefore, environmental challenges have prompted individuals to reflect on the interconnectedness of humans and the natural world. In addition, the progress of human civilization depends on the indispensable use of natural resources [5]. As a result, ecological conservation has increasingly gained appeal as a strategy for safeguarding the environment.

Again, these environmental challenges can be resolved if individuals take personal responsibility for mitigating harmful environmental impacts by increasing their purchases of green goods [6,7]. So, green consumerism plays a crucial role in achieving sustainable development [4]. Hence, it is essential to integrate ecologically sustainable practices throughout all stages of producing goods and services [8]. Green use refers to environmentally conscious use, where consumers consider the environmental effect of purchasing, using, and discarding various green amenities [9]. So, consuming green products (GP) greatly impacts ecological sustainability [1]. In light of this, green consumers greatly aid green transformation. Their green use is essential for a sustainable future and has beneficial implications for the environment, society, and economy [4,10]. When environmentally aware customers become more knowledgeable about sustainable development and cultivate a more worthy attitude, their concern will influence purchasing decisions that greatly impact the ecosystem [11,12]. Therefore, it is crucial to look at the behavioral factors in the rising market to encourage sustainable movement and balance human activities on the environment [1].

Green evaluation is a growing concern in developing nations like Bangladesh since it improves production or food quality, especially for health and the environment [7]. As a result, Bangladesh is trying to adopt green manufacturing and organic farming cultures [7,13]. These initiatives introduce eco-friendly companies to develop and advance the green movement [[14], [15], [16], [17]]. As a result, many businesses have started implementing green manufacturing and marketing methods to satisfy consumer demands and generate long-term profitability [18,19]. Although Bangladesh does not have many green product manufacturers, the trend is growing [7]. On the other hand, recently, consumers have been concerned about the environment and seek green goods and services [20]. Although Bangladeshi consumers still exhibit differing green buying habits [14,15,21], their green behavior (GB) may impact the transformation of social conditions [1]. By sticking to a green lifestyle, people in Bangladesh may make an important move to accept green products [14,15,22,23]. Therefore, it is crucial to identify the factors influencing green behavioral intention (GBI) to promote green consumption and purchase more green products.

Green product manufacturing and consumer behavior are rising daily, especially in urban regions [7]. Although older buyers are the primary purchasers, younger consumers are more interested in purchasing green items [24]. They are considered the society's future [25]. They can help foster a green economy from an ongoing ecological crisis, bring a fresh sustainability phase, and help advance the green motive [1,14]. They consider it their duty to address environmental issues [1,14,26]. So, students are more worried about the future and concerned with social justice, personal safety, and the preservation of the environment [1]. As a result, they are trying to make a lifestyle suitable for themselves and the environment. Therefore, it is crucial for academicians, marketers, and researchers to determine the green aspects that impact undergraduate students' intention to make green purchases which will help to achieve sustainable development.

Several marketing researchers have discussed consumer purchase intentions for green products in various nations [14,[27], [28], [29], [30], [31], [32], [33], [34], [35], [36]]. However, there is a lack of empirical studies in developing nations like Bangladesh [7]. The research on environmental challenges, green products, and environmentally friendly consumer choices is still in the early phases in Bangladesh. A few investigations have looked at the marketing mix in Bangladesh concerning customer perceptions of green marketing [15,[37], [38], [39]]. Using the expanded Theory of Planned Behavior (TPB), few studies on green behavioral intentions have been conducted in Bangladesh [7,15,39]. Again, there has not been enough research on how green factors affect undergraduate students' green behavior. Therefore, this study focuses on undergraduate students to understand their attitudes toward green buying behaviors. So, the goal of the current study is to better understand how green factors influence undergraduate students’ behavioral intentions to buy green products in Bangladesh.

This study's main contributions are addressing a research gap involving green behavioral intentions by employing the expanded Theory of Planned Behavior (TPB) and comparing green factors with prior research. The study adds additional variables, such as green price sensitivity, green product trust, and green product value, to the TPB model. In earlier research, only linear relationships were employed to analyze the link between behavioral intentions and the TPB variables (attitude, subjective norm, and behavioral control). However, this study combines the TPB factors into a single construct, the green TPB perception (GTP), to assess the undergraduates' intentions to behave sustainably. Furthermore, prior research demonstrates that people with high environmental concerns are likelier to have a positive attitude toward GBIs [40]. So, green environmental awareness (GEA) is used in this study as a moderator to ascertain whether or not it affected the connection between the proposed aspects. Additionally, research on the levels of green factors necessary for undergraduate students' green behavior remains unexplored. Therefore, this study employed a necessary condition analysis (NCA) to determine these necessary conditions.

The present investigation makes several important contributions to the corpus of knowledge. First and foremost, incorporating the GBI concept in the research is a unique contribution since it addresses a developing topic crucial in the contemporary day, especially in developing nations like Bangladesh. Second, this study explores GBI by analyzing the effects of TPB constructs (green personal attitude (GPA), green subjective norm (GSN), and green behavioral control (GBC)), green price sensitivity (GPS), green product value (GPV), and young consumers' green trust (GPT) in green products. There have been no previous studies that have looked into this particular set of variables. The study suggests a better understanding of the motives and forces influencing sustainable and environmentally friendly behavior by examining these variables and how they affect undergraduates' GBI. This study can assist in determining the primary drivers of GBI, which can then be utilized to build plans and regulations to support and promote green behavior. Thirdly, this investigation expresses green TPB perception (GTP) as a higher-order construct comprising GPA, GSN, and GBC. The study hopes to get a more comprehensive knowledge of how the combination of TPB factors affects the GBI of undergraduates. Fourthly, the study analyses the moderating effect of GEA on the proposed relationships. The result will help how young students GEA moderate their GBIs. Fifthly, the study employed NCA to determine the green factors necessary for achieving undergraduates' GBI. As a result, the current study concentrates on looking at green factors that impact undergraduates’ GBIs, which will help achieve environmental sustainability.

## 2 Theory and literature review

### 2.1 Theory of planned behavior (TPB)

Analyzing or understanding human behavior, TPB [41] is the best theory [42]. It helps to comprehend the complicated dynamics of young students' green behavior toward green products. Four aspects of TPB help to explain consumers' green buying habits. First, attitude expresses a positive or negative evaluation of green behavior. Second, subjective norms express a personal viewpoint and a societal group of peers about the action to be taken. Third, perceived behavioral control shows the possibility that an activity will be simple or difficult to carry out. Fourth, behavioral intention demonstrates the customer's power to act or decide [41]. Due to TPB's potential for prediction, numerous environmental researchers use it [1,14,42]. According to Ajzen & Madden [43], the logical decision-making process used by people to support the TPB's prescribed behavior is based on consumers' systematic consumption of knowledge. The TPB model may be modified and expanded upon to strengthen its explanatory power by adding additional predictive factors [1,41]. According to Paul et al. [44], the inclusion of additional predictive constructs in the TPB enhances comprehension of the variables that impact green behavioral attributes.

However, this study intents to examine the GBI of undergraduates. For green practices to succeed, consumer behavior change is necessary [1]. The behavior changes pertain to both action execution and the preservation of the environment. It improves the health of the customers [22]. In the environmental setting, "green behavior" refers to direct actions that benefit the environment, such as buying green products. Again, green products and services are non-toxic, long-lasting, and have no negative influence on human health or the environment. Thus, a consumer's intention to engage in green behavior is a plan of action indicating they want to support environmental and public health protection by making green product purchases [45]. People, for instance, act following their prior experiences and routines while purchasing green products or traveling sustainably to save resources [46]. This behavior also entails choosing ecologically responsible methods for finding and purchasing goods and services.

Furthermore, according to research, demographics, financial resources, social standing, values, aptitude, environmental culture, and knowledge affect GBI. Psychographic features, such as attitudes, values, environmental awareness, perceptions, knowledge, and trust, are more responsible than demographic characters for examining GBI [47]. Every person forms certain opinions on a thing and then assesses those opinions in light of their values and preferences in the surroundings [1]. However, environmental protection forces green customers to forgo their demands for high quality [48]. The TPB model is frequently used in research to analyze the preferences and actions of green customers. In accordance with earlier investigations, this study conceptualizes GBI as a unidimensional component [49]. The most recent research on customer behavior toward green products is shown in Table A.

**Table A:** The most recent research on customer behavior toward green products.

Major paper titles	Authors (year) and context	Variables
Intention toward buying green products	[36] (India)	Price sensitivity, government green interventions, green product availability, buying intention
Knowledge and trust matter for purchase intention	[34] (Pakistan)	Environmental knowledge, consumer attitude, green trust, and purchase intention
Examining the factors that affect consumers' purchase intention	[50] (Egypt)	Purchase intention, attitudes, e-WOM, environmental concern, subjective norms, perceived behavioral control, health consciousness, purchase intention.
Green innovation and environmental awareness-driven green purchase	[51] (China)	Environmental awareness, green innovation, green product knowledge, environmental concerns, purchase intention
Drivers in the process of buying green products	[52] (Peru)	Green satisfaction, green trust, green WOM, and green perceived value
I buy green products for my benefits or yours	[35] (China)	Moral obligations, green self-identity, environmental concern, social pressure, green purchase intention, perceived cost, price sensitivity, social pressure
Green purchase intention and behavior	[53] (Philippines)	Awareness of environmental degradation's consequences, green purchase intention, green purchase behavior, attitudes, norms, perceived behavioral control, environmental awareness beliefs, green purchase intention, green purchase behavior
Green consumption behavior	[54] (China)	Pro-environmental awareness, green consumption behavior, consumer perceived cost, policy incentives, and face culture
Organic green purchasing	[55] (India)	Green skepticism, altruistic and egoistic values, perceived consumer effectiveness, environmental involvement, green purchase intention, green attitude, price sensitivity, and environmental protection emotion
Organic food purchase intention	[56] (Portugal)	Environmental concerns, health concerns, perceived quality, attitude, product availability, purchase intention
Purchase intention of organic food products	[50] (Egypt)	Attitudes, environmental concern, e-WOM, subjective norms, perceived behavioral control, health consciousness, purchase intention
Modeling behavioral intention to buy apartments	[57] (Bangladesh)	Green purchase intention, TPB variables, perceived physical quality, access to money, and favorable government policy
Factors influencing generation-Y green behavior	[1] (Nigeria)	Green behavior, green behavioral control, green product trust, green product value, green environmental awareness, green price sensitivity
Purchase intention toward organic food among young consumers	[58] (China)	Attitude, subjective norms, perceived behavioral control, purchase intention, environmental concerns
Factors influencing green product purchase intention	[14] (Bangladesh)	Green purchase intention, attitude, environmental concern, willingness to pay, perceived moral obligation, intention
Consumers' intention to green purchase decision	[7] (Bangladesh)	Environmental concern, green perceived quality, green awareness of price, green willingness to purchase, green future estimation, green purchase decision
Green consumption behaviors	[59] (China)	Environmental awareness, firm sustainability exposure, and green consumption behaviors
Making green food choices	[33] (China)	Respondent characteristics, purchase intention, willingness to pay
Consumer behavior toward organic food	[60] (Brazil)	Socio-economic and demography, motivation, perception, attitude
Purpose of purchasing authentic green furniture	[32] (China)	Environmental consciousness, subjective norm, perceived behavior control, physical health concern, purchase intention, experience, and attitude
Explaining consumer purchase behavior for organic milk	[61] (Italy)	Trust, green self-identity, purchase behavior
Consumers' intentions toward purchasing green food	[62] (China)	Attitude, perceived behavioral control, face consciousness, confidence, purchase intention
Factors influencing organic food purchase intention	[63] (Tanzania and Kenya)	Subjective norms, personal attitudes, health consciousness knowledge, PBC, purchase intention
Consumer behaviors in the green hotel	[64] (Pakistan)	Personal norms, behavioral intention, environmental consciousness, green consumer behavior
Consumers' green purchasing behavior	[27] (China)	Attitude, green products information, green (product quality and purchase behavior), environmental consciousness
Consumers' actual purchase behavior	[15] Bangladesh	Green product availability, information and price, purchase intention
Purchase behavior toward green brands	[65] (Taiwan)	Green perceived value, quality, risk, information costs, purchase intentions
Purchase intention toward green apparel products	[66] (USA and China)	Environmental knowledge, subjective norm
Green emerging market	[67] (Vietnam)	Attitudes, knowledge, norms, rational, moral, self-identity and perceived barriers, emotional and self-identity
Consumer behavior toward organic foods	[68] (Australia)	Trust, healthism, hedonism, experiences, and purchase intention

### 2.2 Green TPB perception (GTP)

Several studies have suggested that TPB factors (attitude, subjective norms, and perceived behavior control) influence consumers' intention to make purchases [1,14,50,56,63]. An individual's attitude can be used to assess how well a specific activity has been performed. According to Ajzen [41], people are more likely to participate in a certain activity if they have a good opinion of the potential results. In this study, “green personal attitude” (GPA) refers to how environmentally conscious young customers assess green products before deciding which ones to buy. A customer's GPA about green products contributes to the growth of a positive GBI [14,37,44,57]. This positive relationship is also supported by other fields, for example, eco-friendly packaging [69], green hotel visit intention [61], apartment purchasing intentions [57] and predicting organic foods [70].

Ajzen [41] asserts that behavioral control explains whether a certain behavior is considered simple or complex to carry out. According to certain research [44], these control elements are used as a metric to assess customer intention and behavior. It is claimed that customers' decisions not to buy green products are influenced by high-cost sensitivity and product shortage [71]. Again, barriers like effort, product cost, and time constraints impact consumer GBI [1]. In order to achieve green product consumption, these obstacles need to be overcome [72]. The stronger the green behavioral control (GBC), the greater the propensity of young green consumers to buy green products. Earlier scholars found a significant association between GBC and GBI [1,36,57,70].

Subjective norm implies that others would agree with a choice to consume or refrain from doing so. That is, whether or not a person engages in the behavior in an issue is determined by how they perceive the pressure from others [70,[73], [74], [75]]. Sometimes, green consumers may be influenced by family members and friends to purchase green products because young people believe in their peers for their green behaviors. This perception may persuade someone to buy green products. Earlier studies found a significant association between green subjective norms (GSN) and behavioral intention [50,57,70,76].

However, sufficient evidence supports that the TPB characteristics play a significant role in forming customer perceptions of green products, influencing their GBIs. Furthermore, according to TPB theory, a person with these characteristics is more driven to demonstrate their behavioral intended use. In earlier studies, individual association of TPB constructs and behavioral intention has been investigated. But, their combined impact on GBI has not been studied in the context of undergraduates. Therefore, this study seeks to explore the combined impact of these parameters (GTP) on GBI. So, the proposition is-

H1 GTP has a significant impact on undergraduate students' GBI.

### 2.3 Green price sensitivity (GPS)

One barrier to using eco-friendly items is GPS [77]. The price elasticity of demand is a popular method for determining pricing in economics. It claims that certain customers will pay less for goods if offered a cheaper substitute and option. According to Ogiemwonyi [1], GPS is a demand shift resulting from a price adjustment. Some people, for instance, are hesitant to spend a penny more for any goods, especially if a reduced price is offered for an item with environmental benefits. GPS is the degree to which consumer demand fluctuates when a green product's price also does. It has favorable effects on the environment. The degree of significance customers attach to price compared to other purchase factors affects how sensitive consumers are to price. Some customers prioritize product quality over price, making them less sensitive to pricing [78]. On the other hand, a customer who is more concerned with pricing is prepared to forego quality [79]. As a result, price sensitivity affects individual decision-making and behavioral intention [7,80].

However, consumers and individuals have different levels of price sensitivity. Certain customers spend more than others for green products and services. According to a previous survey, 67% of American customers are prepared to spend an extra 5–10% on items that are sustainable and environmentally friendly (as mentioned [1]). Eco-conscious customers are prepared to pay a higher price for green products [81]. On the contrary, a few investigations in the literature contend that consumers are more willing to pay more attention to price than safeguarding the environment [82]. When examining the concept of pricing as it relates to human behavior, customer willingness to spend for a product is a crucial consideration. According to earlier research, GPS is important when buying green products [15,55,83]. It positively affects green behavior [1] and has negative effects [35,36]. The investigations suggest that GPS affects GBI because of the distinctiveness of price sensitivity on green behavior [35,55]. So, the proposition is-

H2 GPS has a significant impact on undergraduate students' GBI.

### 2.4 Green product trust (GPT)

According to earlier studies on green marketing, customer trust in green products favorably affects their purchasing decisions [1,84]. Studies in social psychology describe trust in terms of the goodwill and reliability of other people. Regarding reliability, we mean how much we can trust people's words, actions, and speech. Goodwill, on the other hand, refers to consideration for the purposes and well-being of both sides in the achievement of a shared interest [85]. Green customers feel less anxious and uncertain when they strongly trust green products. It increases the reliability of the supplier of the goods and services. Four criteria—personality-oriented, experience-based, cognition-based, and effect-based—are used to develop GPT [86]. A person's daily habits and personality are known as personal-oriented attributes. "Experience-based dimension" refers to a consumer's total appraisal of their interactions with a service operator. The effects of a consumer's engagement with a salesperson on their perception are directly defined by cognition-based variables. The effect-based aspect is held by how customers see a company in the context of outside effects from third parties. Therefore, GPT may have an impact on consumer decision-making during the buying process.

Furthermore, according to Karatu & Mat [87], GPT refers to a consumer's readiness to rely on a product due to their conviction that it would satisfy their needs in terms of the environment. Similarly, in the light of the TPB model, a person's tendency to trust is shown by their behavior. Furthermore [74], stated that it is a direct precursor of green behavior. According to many scholars, GPT significantly impacts GBI [34,52,85,88,89]. So, the proposition is-

H3 GPT has a significant impact on undergraduate students' GBI.

### 2.5 Green product value (GPV)

According to the marketing literature, a company's products and services have benefits and drawbacks [90]. The products which have sustainable value benefit the environment. GPV is the customer's general assessment of the final profit on expenses or price of a good or service [91]. Consumers' environmental desires, needs for sustainability, and expectations are used to evaluate what is accepted and delivered. Yaacob & Zakaria [92] claimed that green buyers favor sustainable goods to protect the environment. For example, direct advantages raise environmental issues, and consumers are encouraged to buy green products because of their health benefits. According to some works on GPV, it can predict green behavior [1,93] and consumers' intention to make green purchases [83]. The impact of sustainable goods and their values on customer purchasing behavior has been recognized in several studies [94,95]. In contrast, several investigations have shown conflicting results [96].

Again Chen & Chang [93], stated that manufacturers of green products design their goods with environmentally friendly qualities. These kinds of services offer high-value characteristics that draw green customers. GPV reduces mistrust and improves customer buying habits [1]. Green consumers value their green products and embrace them in their social surroundings. So, the proposition is-

H4 GPV has a significant impact on undergraduate students' GBI.

### 2.6 Green environmental awareness (GEA) as a moderator

According to Lee [49], GEA refers to how well people comprehend environmental concerns as a matter of facts, ideas, and connections to other aspects of the environment. A more environmentally conscious customer is likelier to seek and purchase a green product. Three essential components are required for a customer with the correct intentions to be aware of and buy green products. These include health awareness, economic harmony, and environmental preservation [58,97]. The present research suggests that GEA may favor how young customers behave in terms of being environmentally friendly. It demonstrates awareness when people buy green products and promote a greener lifestyle to save the environment. Earlier research stated that green customers choose sustainable items to show their care for the ecosystem [58,98].

Prior research on environmental awareness has concentrated on environmental concerns, including conserving energy and pollution [99]. In contrast, current studies focus on total GEA [1,51,53,54,100,101]. Concerns about the environment are prevalent among society and young customers. As a result, this study explores the significance of researching moderator and predictive variables in GBI. Prior studies have used various scales to assess consumer environmental awareness concerning various issues [102]. The New Environmental Paradigm (NEP) is a part of it [103,104]. In many study settings, NEP scales are used to determine environmental awareness parameters, for example, tourism, marketing, and behavioral research [1,97,105]. Using the expanded TPB model [97,105], recommend generic GEA as a framework for measuring green consumption. Again, environmental awareness affects the results of the TPB framework [59]. Therefore, this study views awareness as crucial to estimating young consumers' environmental behavior.

Nowadays, educational institutions are concerned about environmental issues. So, GEA is now a part of education and learning. Higher education institutions teach their students to become environmentally friendly. According to Zorić & Hrovatin [106], such knowledge affects an undergraduate's propensity to engage in pro-environmental actions. The GEA research on young consumers' environmental behavior in Bangladesh is inadequate. This work evaluates GEA using a single dimension [107,108]. In several earlier research, it has been demonstrated that environmental awareness has a favorable impact on pro-environmental behavior [1,51,87,109]. So, the propositions are-

H5 GEA moderates the association between GTP and GBI for undergraduate students.

H6 GEA moderates the association between GPS and GBI for undergraduate students.

H7 GEA moderates the association between GPT and GBI for undergraduate students.

H8 GEA moderates the association between GPV and GBI for undergraduate students.

## 3 Research methodology

### 3.1 Propose model

**Fig. 1:**
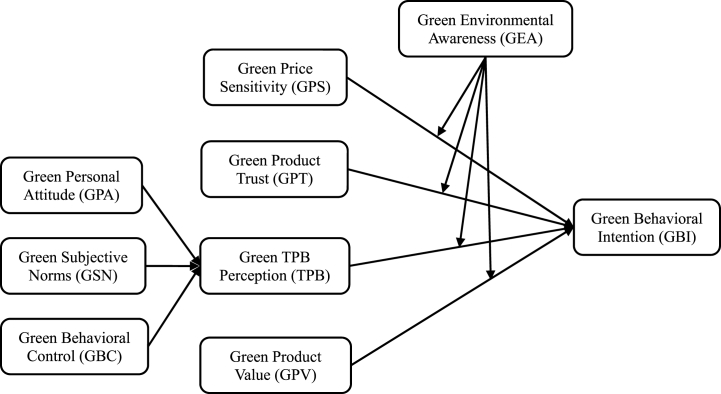
Conceptual framework.

Fig. 1 represents the proposed research model.

### 3.2 Target respondents

The study was conducted in Dhaka City, Bangladesh. Undergraduate students from various private universities were the respondents of this work. The research methodology used was quantitative. According to earlier research [44,110], educated consumers who reside in cities are more anxious about green products because they are aware of and knowledgeable about them. Again, young consumers are more concerned about environmental issues, and they are the key to bringing about the desired change in green product purchases. So, this study used undergraduate young green consumers' information to meet study objectives. A non-probability convenience sampling was employed to gather the data [111]. This approach was chosen because it was able to gather high-quality data [112]. It is cost-effective and capable of controlling the different types of responses [113]. Respondents were asked a common question about their previous experience purchasing eco-friendly products, such as organic food, recycled toilet paper, LED lights, reusable cups, rechargeable batteries, energy efficiency products etc. If they said yes, the researcher requested them to complete the questionnaire.

### 3.3 Required sample size

The study employed G*power software (v3.1.9.4) to determine the minimum sample size [114]. The researcher used an effect size of 0.05 and a statistical power of 0.95. The minimum required sample size was 262. Participants in the survey provided a total of 427 replies. The researcher removed the incomplete responses. For further evaluation, 382 valid responses were utilized. As a result, the sample size was enough for the statistical assessment.

### 3.4 Instrumentation

The indicators used in the survey have been verified in prior research. Several changes have been made to fit the objectives and purposes of the present investigation. A pilot survey with 40 participants was conducted before the main study. It ensures the validity and usefulness of the replies [115]. A seven-point Likert scale was utilized in this research (strongly disagree = 1 to strongly agree = 7). GPA comprises six items [63], and GSN comprises five [63,116,117]. GBC (5 items), GPS (5 items), GPV (5 items), GEA (5 items), GPT (5 items), and GBI (6 items) are adapted from earlier work [1]. Appendix A represents the measurement items.

### 3.5 Green TPB perception (GTP) as a higher-order construct

In this study, GTP was proposed as a higher-order construct. It was a second-order reflective-formative construct. It consisted of three lower (first) order constructs (i.e., GPA, GSN, and GBC). These constructs were reflective. From a methodological perspective, higher-order constructs were suggested. They simplify the model by lowering the number of suggested relations, which makes it more concise [118]. Higher-order construct also aids in minimizing collinearity problems [119]. Additionally, it facilitates the interpretation of outcomes and helps produce trustworthy and accurate empirical findings [118].

The study followed [120] guidelines for reflective or formative measurement model specification. The higher-order construct, GTP, consisted of three lower-order constructs. These variables had their own set of indicators and distinct theoretical meanings. Their theoretical meanings were reflected by their measures. So, the lower-order constructs were estimated by their indicators, and the higher-order construct was measured with these lower-order constructs by applying a disjoint two-stage approach [121].

There were mainly two reasons for proposing GTP as a higher-order construct. First, a number of recent research employed the TPB's suggested components (attitude, subjective norms, and behavioral control) to examine the impact on behavioral intention [1,14,63]. The structural model had three different relations when using three independent factors. The number of possible relations was decreased when these three distinct factors were combined. Second, the idea of "TPB concepts" was put out in several earlier research, where it was examined as a multifaceted concept using all three suggested aspects [1,14,63]. These investigations also inspired combining these dimensions into a single higher-order construct.

### 3.6 Respondents' profile

Study findings revealed that the average age of the respondents was 22.58 years (standard deviation 1.147 years). Their minimum age was 19 years, and the maximum was 24 years. Most undergraduates were male (57.90%), and the rest were female (42.10%). Again, most of them were 3rd-year students (52.10%), followed by 2nd year (26.70%), 4th year (12.00%), and 1st year (9.20%). See Table 1.

**Table 1:** Undergraduates demographics.

Variables	Categories	Frequency	Percent
Gender	Male	221	57.90
	Female	161	42.10
Academic year	1st year	35	9.20
	2 nd year	102	26.70
	3rd year	199	52.10
	4th year	46	12.00

## 4 Data analysis and results

The study aims to look into the variables that affect undergraduate students' GBI. In this work, partial least square structural equation modeling (PLS-SEM) has been used. The PLS-SEM can simultaneously handle higher-order constructs [119,122] and moderator evaluation [123] in a single framework. The study proposed the "GTP" as a reflective-formative higher-order construct. Therefore, PLS-SEM is a good option for model evaluation. Another justification for using PLS-SEM is that the study's major objective is predicting the key constructs using a sophisticated research model [124,125]. This study utilized SPSS 22, SmartPLS (v 3.3.5), and R software for various estimations. For PLS-SEM data analysis, a two-stage procedure has been used [124,126]. Step one involved evaluating the measurement model, and Step two involved evaluating the structural model.

### 4.1 Common method bias (CMB)

According to Avolio et al. (1991), CMB is frequently observed in investigations that gather responses from a single source. It could be problematic for quantitative studies that rely on information provided by participants [127]. The structural path is impacted by CMB [128], which declines reliability [129]. This study employed Kock's [130] collinearity test for checking CMB. It was discovered that the latent components' variance inflation factor (VIF) estimates are under the 5.0 cut-off. So, CMB is not a problem for this investigation.

### 4.2 Measurement model

#### 4.2.1 Assessment of reflective constructs

This study employed factors loadings (λ), construct reliability, convergent validity, and discriminant validity to assess the adequacy of the model [21]. Table 2 demonstrates that λ values of the lower-order constructs exceed the cut-off value of 0.70. A composite reliability (CR) and Cronbach's alpha (α) score of > 0.7 indicate a substantial internal consistency, while an average variance extracted (AVE) value > 0.5 indicates the presence of convergent validity [131]. Again, the study used the Fornell & Larcker [132] and Heterotrait-Monotrait Ratio (HTMT) criteria to evaluate the discriminant validity. As displayed in Table 3, all the HTMT ratio values are considerably lower than the cut-off point of 0.85 [128]. Table 2 and 3 contain the outcomes of the measurement model evaluation.

**Table 2:** Assessment of construct validity.

Constructs	Items	λ	α	CR	AVE
Green Personal Attitude (GPA)	GPA1	0.868	0.919	0.937	0.712
	GPA2	0.844			
	GPA3	0.851			
	GPA4	0.833			
	GPA5	0.813			
	GPA6	0.853			
Green Behavioral Control (GBC)	GBC1	0.828	0.926	0.944	0.771
	GBC2	0.920			
	GBC3	0.894			
	GBC4	0.866			
	GBC5	0.881			
Green Subjective Norms (GSN)	GSN1	0.855	0.918	0.939	0.754
	GSN2	0.888			
	GSN3	0.873			
	GSN4	0.857			
	GSN5	0.868			
Green Price Sensitivity (GPS)	GPS1	0.852	0.915	0.937	0.747
	GPS2	0.888			
	GPS3	0.872			
	GPS4	0.845			
	GPS5	0.864			
Green Product Trust (GPT)	GPT1	0.869	0.918	0.938	0.753
	GPT2	0.863			
	GPT3	0.870			
	GPT4	0.871			
	GPT5	0.866			
Green Product Value (GPV)	GPV1	0.894	0.932	0.948	0.785
	GPV2	0.906			
	GPV3	0.869			
	GPV4	0.883			
	GPV5	0.878			
Green Environmental Awareness (GEA)	GEA1	0.882	0.926	0.944	0.771
	GEA2	0.894			
	GEA3	0.862			
	GEA4	0.866			
	GEA5	0.887			
Green Behavioral Intention (GBI)	GBI1	0.919	0.956	0.965	0.820
	GBI2	0.914			
	GBI3	0.913			
	GBI4	0.914			
	GBI5	0.873			
	GBI6	0.898

**Table 3:** Assessment of discriminant validity.

Fornell-Larcker Criterion								
	GBC	GBI	GEA	GPA	GPS	GPT	GPV	GSN
GBC	**0.878**							
GBI	0.735	**0.905**						
GEA	0.672	0.786	**0.878**					
GPA	0.613	0.774	0.682	**0.844**				
GPS	0.628	0.793	0.682	0.670	**0.864**			
GPT	0.596	0.775	0.693	0.750	0.636	**0.868**		
GPV	0.633	0.789	0.691	0.736	0.684	0.715	**0.886**	
GSN	0.639	0.796	0.701	0.671	0.710	0.738	0.715	**0.868**
HTMT Ratio								
	GBC	GBI	GEA	GPA	GPS	GPT	GPV	GSN
GBC								
GBI	0.782							
GEA	0.726	0.834						
GPA	0.665	0.825	0.738					
GPS	0.681	0.847	0.741	0.728				
GPT	0.645	0.826	0.750	0.815	0.692			
GPV	0.681	0.836	0.743	0.794	0.741	0.771		
GSN	0.692	0.849	0.759	0.728	0.773	0.804	0.771	

Note: The above matrix's diagonal values (bold) represent the square roots of AVEs, whereas the off-diagonal values represent correlations between the latent components.

#### 4.2.2 Assessment of the formative construct

The current work hypothesized GTP as a reflective-formative higher-order construct. To examine the reflective-formative higher-order construct, the researcher followed a disjoint two-stage approach [133,134]. The latent variable scores for the lower-order constructs were computed during the first stage. In the second stage, the latent variable score derived from the PLS algorithm was utilized to compute the weight and significance. The formative construct was determined by analyzing the indicators' VIF values and weight. Table 4 displays the findings. All measures had a VIF < 5.0, indicating that collinearity was not a significant issue [130,135]. A 5000-resample bootstrapping method was employed to evaluate the significance of the weight. Study outcomes demonstrate that weights were statistically significant at a p-value of less than 0.001. So, the results exhibit the proportional contribution of the reflective constructs toward forming a reflective-formative higher-order construct.

**Table 4:** Higher-order construct (HOC) assessment.

HOC	LOCs	VIF	OW	t-values	95% BC-CIs
Green TPB Perceptions (GTP)	GPA	2.302	0.406	6.741*	[0.324, 0.520]
	GSN	2.155	0.452	8.883*	[0.336, 0.554]
	GBC	2.007	0.272	5.303*	[0.181, 0.371]

Note: OW = Outer weight, LOC = Lower-order construct, HOC = Higher-order construct, BC-CI = Bias corrected confidence interval, *p < 0.001.

#### 4.2.3 Assessment of the structural model

**Fig. 2:**
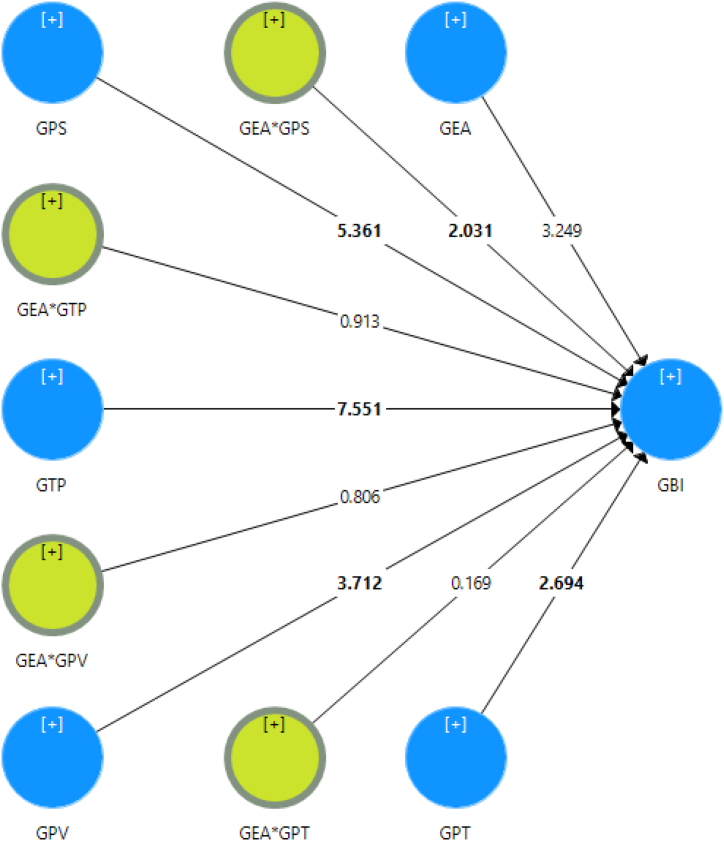
Results of the structural model.

After validating the measurement model, it was necessary to examine the structural model to validate the proposed hypotheses [131,136]. The structural model was evaluated utilizing path coefficients (β) and R^2^. The outcomes confirmed that all the direct hypotheses are supported. The study results revealed that GTP (β = 0.420, p < 0.001), GPS (β = 0.204, p < 0.001), GPT (β = 0.122, p < 0.01), and GVT (β = 0.137, p < 0.001) are significant predictors of GBI. Therefore, the results validated all the direct hypotheses (H1, H2, H3, and H4). See Fig. 2 and Table 5.

**Table 5:** Assessment of direct and moderation effects.

Hypotheses		β	t-values	p-values	Supported
	*Direct effects*				
H1	GTP - > GBI	0.420	7.551	0.000	Yes
H2	GPS - > GBI	0.204	5.361	0.000	Yes
H3	GPT - > GBI	0.122	2.694	0.008	Yes
H4	GPV - > GBI	0.137	3.712	0.000	Yes
	*Moderation effects*				
H5	GEA*GTP - > GBI	−0.045	0.913	0.363	No
H6	GEA*GPS - > GBI	0.099	2.031	0.045	Yes
H7	GEA*GPT - > GBI	0.009	0.169	0.867	No
H8	GEA*GPV - > GBI	−0.036	0.806	0.422	No

**Fig. 3:**
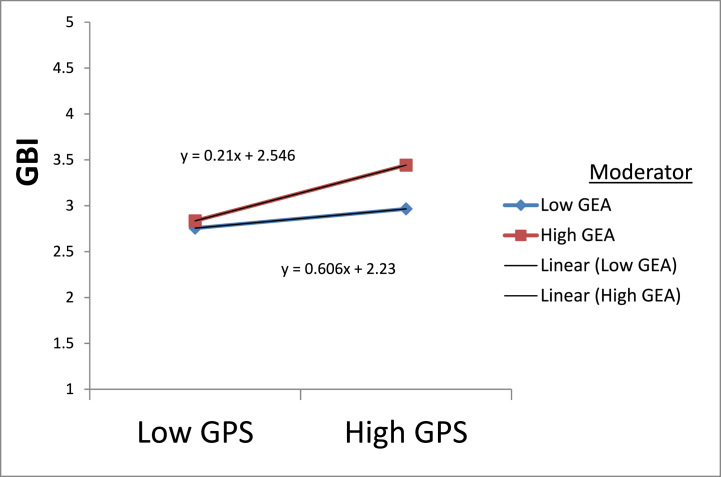
Moderation effects of GEA.

Again, according to the moderation analysis, GEA moderated the association between 10.13039/100011109GPS and GBI as β = 0.099 and p < 0.05 and supported hypothesis H6. That means GEA strengthens the positive relationship between GPS and GBI. See Fig. 3. However, the rest of the other relationships of moderation were found insignificant; hence, hypotheses H5, H7, and H8 were not supported. See Table 5.

#### 4.2.4 Assessment of the explanatory power

The coefficient of determination (R^2^) was calculated to assess the structural model's adequacy. The R^2^ value for the GBI was 0.844. Therefore, all the independent variables GTP, GPS, GPT, and GPV explained 84.40% of the variation of GBI. So, the model thus has a strong explanatory power. The goodness of fit index is another metric to assess model fit. In this study, the method Henseler et al. [137] recommended was employed to evaluate standardized root mean square residuals (SRMR), using a maximum threshold value of 0.080. With an SRMR value of 0.038, this study demonstrates significant goodness of fit.

### 4.3 Necessary condition analysis (NCA)

**Fig. 4:**
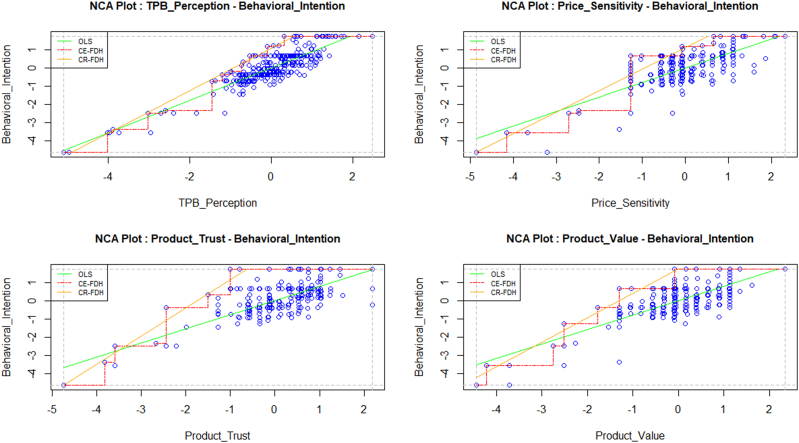
Results of NCA analysis- GTP, GPS, GPT, GPV, and GBI; OLS regression line; Scatter plot, CR-FDH ceiling line.

This work employed NCA to assess the degree of necessity of GTP, GPS, GPT, and GPV required for young consumers' GBI. This analytical technique helps researchers determine the extent of a necessary condition for the happening of the dependent variable. But it is not providing sufficient conditions. In order to measure the degree of necessary conditions, this approach calculates the ceiling line. The ceiling line splits the entire space neatly into two sections, including and excluding observations [138]. There are primarily two techniques for determining the ceiling line. The first one is called-ceiling envelopment with free disposal hull (CE-FDH), and another one is called ceiling regression with free disposal hull (CR-FDH). For continuous data sets, the second technique is used [138]. Table 6 and Fig. 4 present the NCA package's findings. For details on NCA, check [138].

**Table 6:** Bottleneck table.

	CE-FDH				CR-FDH			
GBI	GTP	GPS	GPT	GPV	GTP	GPS	GPT	GPV
0	NN	NN	NN	NN	3.0	NN	0.3	NN
10	14.3	9.8	13.4	3.4	10.1	7.4	6.2	2.5
20	27.3	30.0	16.7	24.9	17.3	14.9	12.1	9.5
30	27.3	30.0	16.7	24.9	24.5	22.5	18.0	16.4
40	48.2	50.0	33.3	28.6	31.7	30.	23.9	23.4
50	48.2	50.0	33.3	28.6	38.9	37.5	29.8	30.4
60	48.2	50.0	33.3	39.4	46.4	45.1	35.7	37.3
70	57.7	50.0	46.7	46.4	53.2	52.3	41.6	44.3
80	60.4	50.0	54.0	46.4	60.4	60.1	47.5	51.2
90	66.0	66.7	54.0	64.3	67.6	67.7	53.4	58.2
100	71.4	76.9	54.0	64.3	74.8	75.2	59.3	65.1
*effect size, d*	0.435	0.432	0.331	0.342	0.389	0.375	0.298	0.305
*p-value*	0.000	0.000	0.000	0.000	0.000	0.000	0.000	0.000
*c-accuracy*	100%	100%	100%	100%	96.10%	94.00%	98.20%	97.40%

The ceiling lines and bottleneck tables are estimated using the NCA application. The bottlenecks display the necessary GTP, GPS, GPT, and GPV levels for GBI. The degree of all necessary conditions is calculated in Table 6 using their bottlenecks. The bottlenecks represent a percentage of the range of observed values. In this study, 0% represents the lowest value, and 100% represents the highest value. Findings from the bottleneck revealed that these components had differing degrees of necessity. Dul [138] recommended that the effect size ranges from 0.0 to 0.1 to be small, 0.1–0.3 to be medium, 0.3–0.5 to be large, and more than 0.5 to be extremely large.

The GTP (d_CR-FDH_ = 0.389), GPS (d_CR-FDH_ = 0.375), and GPV (d_CR-FDH_ = 0.305) effect sizes are within a large range, and GPT (d_CR-FDH_ = 0.298) is the withing medium range for GBI, which characterizes a significant effect. According to the NCA, GTP has the highest impact on GBI. Again, CR-FDH ceiling line results revealed that at 10% of the GBI, all the independent variables are necessary at various levels, for example, GTP (10.1%), GPS (7.4%), GPT (6.2%), and GPV (2.5%). Again, for 80% percent of GBI, all four constructs were necessary conditions (GTP = 60.4%, GPS = 60.1%, GPT = 47.5%, and GPV = 51.2%). See Table 6.

## 5 Discussion

The research investigates the green factors affecting undergraduate students' behavior toward green products. The outcome reveals that each factor - GTP, GPS, GPT, and GPV significantly impacts students' GBI. The findings are shown in Table 5. The results suggest that GTP has a significant positive effect on GBI, and it is the best predictor among other variables based on the beta coefficient (*β = 0.420*). Additionally, the results showed that GTP is crucial to students' GBI improvement. These results increase our knowledge of the relative impact of GTP on GBI in the green products setting. These new details help us better comprehend how GTP influences GBI. The positive association between GTPs and GBI is consistent with the earlier work [1,14,45,63,139]. Young consumers may have power over their activities to demonstrate green behavior and acquire green products, trusting in green product credibility, which might account for the significant result.

Once more, the findings demonstrate that the impact of GPS on young students' environmental behavior is positive. The findings concur with earlier research on green products [1,140]. Regarding Bangladeshi customers' low-income level and their strong price sensitivity, undergraduate students nevertheless purchase and like green products. Despite the influence of price sensitivity, the investigation demonstrates that students will pay more for a high-quality product. Green product manufacturers typically demand more for their goods. Customers are price sensitive; occasionally, they will buy green products, but not if they are more expensive. Nevertheless, price sensitivity might be a significant obstacle for some consumers if no discounts or promotions are available. As a result, value for money is obtained while also making the quality and reliability of products acceptable. Therefore, if the price exceeds what the buyer would expect, it diminishes the effect of young students' green behavior and widens the gap between green and conventional purchasing.

The third hypothesis revealed a favorable effect of GPT on students' GBI. It shows that young customers believe green products are typically trustworthy and that their promises live up to their expectations. In light of this, the study's findings indicate that young customers willingly receive green products. According to the results, consumer trust is rising, which suggests that more young people are buying eco-friendly goods. The results are analogous to earlier research [1,88] but contradict other studies [83]. According to prior research [6], consumers' mistrust of the ethical promises of green products deters their purchase intentions. Furthermore, GPV has a considerable impact on undergraduates' GBI. The GPV's significant impact shows how well green products perform environmentally and compare well in terms of value with traditional goods. The findings concur with other studies [1,141], emphasizing the significance of GPV and green behavior as determining factors for buying green products.

In addition, the study tried to look at how GEA affected the suggested research model. Individual green behaviors are significantly influenced by GEA [142]. Additionally, environmental deterioration forces individuals to adopt a green lifestyle. As a result, pupils also think green policies can save the earth. The study's findings indicate that GEA is crucial to the interaction between GPS and GBI. It suggests that if a consumer has a high GEA, GPS will have a higher impact on GBI. It highlights the significance of consumer knowledge for a better environment in influencing students' green purchasing decisions. It demonstrates how environmental knowledge of green products positively impacts GBI. The results are consistent with other research on green behavior-related environmental issues [46,143]. Therefore, GEA is crucial because young consumers feel they have to safeguard the environment, which is deteriorating due to environmental damage. They favor promoting a healthy lifestyle that is favorable to the environment. However, other moderation effects are insignificant and need further investigation.

Additionally, the NCA reported that GTP, GPS, GPT, and GPV had varying levels necessary to effectively implement GBI. NCA also found that GTP is the best predictor of GBI of undergraduate students, similar to the SEM results. These conclusions have several consequences for theories, investigation, and application.

## 6 Conclusion and implications

The study's primary goal is to examine the green factors affecting undergraduate students' green behavior toward green products. The extended TPB model was adopted for this study. The study significantly contributes by developing a unique paradigm considering environmental and TPB factors. According to various studies, young customers are the next generation of green marketing; thus, policymakers need to grasp this [1,14,15,144]. The present research concerns young students' GBI. As a result, this sheds further light on the constraints of green marketing research, which emphasizes the desire to purchase and adds to the viewpoint on green behavior. The study also offers an important understanding of how green factors like GTP, GPS, GPT, GPV, and GEA affect the environmental behavior of young consumers.

According to previous research in Bangladesh, young green people are ready to buy green products [7,14,15]. However, encouraging the use of green products influences young pupils' green behavior and boosts the purchasing of green products. The results revealed that GEA helps to increase young consumers' perceptions of green behavior. It is appreciable to imagine pupils acting sustainably. The important discoveries aid managers and decision-makers in comprehending the basic psychology of the customers they aim to target, particularly those factors influencing green behavior. Policymakers may develop a practical marketing strategy for the Bangladeshi market once they realize how important green behavior among undergraduates is to the success of green marketing. Again, university authorities should be aware undergraduates about green products' environmental and health advantages because these benefits are the primary drivers of green product purchases. Young, eco-conscious customers are curious to contribute responsibly to society. They are craving to solve environmental problems and current difficulties [1]. So, engaging youngsters in green behavior helps them develop a positive attitude toward green behavior. Therefore, authorities should highlight to undergraduates how unique green products are and how they vary from conventional and non-green items. As a result, young customers get more trusting of green products. It motivates them to adopt green behavior, which, in turn, helps in achieving environmental sustainability.

In recent times, Bangladeshi customers have been aware of eco-friendly items but are not particularly concerned with green marketing and behavior [7]. Once individuals know this, their interest in behaving green and making eco-friendly purchases considerably increases. These findings indicate that the marketer must create a customer base for green products. It is also essential for marketers to give customers the necessary information about how to behave green and use green goods. Therefore, the business and marketer should start appropriate efforts to advertise green goods and marketing. They can educate customers about green products by utilizing green marketing strategies, including eco-friendly branding, advertising, and eco-labels. They can implement green advertising highlighting the product's environmental advantages, encouraging a sustainable lifestyle, enhancing the brand's image, and minimizing the shortcomings typical of green products [145]. In addition, eco-labels are a crucial marketing and advertising tool that can raise consumer awareness of environmental issues, boost a product's sales and brand quality, and urge producers to consider how their goods' environmental impact. According to a survey, 70% of customers' purchase decisions are influenced by environmental messaging in advertisements and product labels [7,146]. 10.13039/100014337Furthermore, eco-branding supports the growth of the green market and the difficulty of changing production [147]. Therefore, marketers should emphasize accurate and clear information about eco-friendly goods or services using eco-labels to promote consumer acceptance of eco-friendly goods and raise awareness of such goods or services.

The developing countries like Bangladesh, farmers and suppliers may produce organic food without utilizing the pesticide formalin or other combined chemicals. Several stores, like shopping malls and supermarkets, can start the growing practice of selling green items. A firm can demonstrate its strong commitment to society through effective environmental campaigns. For example, it can promote energy-saving ideas and raise customer knowledge of green products. Governments, health organizations, and environmental advocacy groups can use direct persuasion to promote environmentally safe products through various communication platforms or methods, e. g., websites or Facebook. Continuous education about eco-friendly products can positively influence consumers' green behavior and decisions to switch from conventional food products and urge them to buy green alternatives. So, the key to sustainable and green consumption is consumer and conscious consumer behavior.

In contrast to other growing nations, Bangladesh has a challenge because of price sensitivity. Therefore, in order to facilitate price reduction, the government should promote green product stakeholders and marketers to manufacture green products domestically. Externally produced green products are pricey. The problem of green product prices should be investigated by policymakers, who should also work to increase green product value by highlighting the green product's benefits to young people's health and the environment. Product scarcity is another hurdle that drives prices and discourages young green consumers. Therefore, governments should make green products accessible to the general public, encourage consumer preference in choosing goods at fair prices, and foster consumer confidence. Government agencies may pressure businesses and marketers to develop green products. Again, producers and policymakers in Bangladesh need to manage the country's steadily rising levels of consumption and production and successfully guide them toward sustainability. Consumers are now aware of the importance of environmental and food security. Therefore, raising consumer awareness and promoting green consumerism will increase green behavior and buying decisions. This empirical quantitative study will assist producers and marketers in comprehending how consumers currently perceive, need, or want safer, better, and healthier product production. Thus, it ensures meeting pertinent sustainable development goals and targets, reducing pollution, and safeguarding the environment. Therefore, farmers, manufacturers, merchants, and governmental organizations must collaborate to ensure green production that appeals to customers to meet these goals.

### 6.1 Limitations and future directions

The present investigation looks at the green factors influencing young consumers' green buying choices and the moderating impact of GEA on these associations. However, this study has several limitations. A convenience sampling strategy is used in the study to gauge young consumers' environmental behavior. However, this method does not adequately account for Bangladesh's whole population. Again, this work was concentrated only on undergraduate students. Therefore, future studies should consider the representativeness of other age groups. The research concentrates on one growing country. In subsequent studies, young consumers in developed and emerging nations should be compared and contrasted using a cross-cultural comparative approach. Additionally, this study considers only linear relationships among variables; future studies may consider non-linear relationships.

## Ethical statement

The researcher confirms that all research was performed in accordance with relevant guidelines/regulations applicable when human participants are involved (e.g., Declaration of Helsinki or similar).

## Funding

This research did not receive any specific grant from funding agencies in the public, commercial, or not-for-profit sectors.

## Informed consent

Informed consent was obtained from all participants in this study.

## Data availability statement

The data that has been used is confidential.

## CRediT authorship contribution statement

**Sanjoy Kumar Roy:** Writing – review & editing, Writing – original draft, Visualization, Methodology, Investigation, Formal analysis, Data curation, Conceptualization.

## Declaration of competing interest

The author declares no conflict of interest.

## Figures and Tables

**Table:** 

Green personal attitude (GPA)
1. I think that purchasing green products is a good idea.
2. I think that purchasing green products is interesting.
3. I think that purchasing green products is important.
4. I think that purchasing green products is beneficial.
5. I think that purchasing green products is wise.
6. I think that purchasing green products is favorable
**Green subjective norms (GSN)**
1. My family thinks that I should buy green products rather than non-green products.
2. My friends expect me to buy green products.
3. People I value, such as my teachers, think I should buy green products.
4. Media influences me to buy green products.
5. Most people I value would buy green products rather than non-green products.
**Green behavioral control (GBC)**
1. I believe it's entirely my decision to buy a green product.
2. I believe I can pay more to buy a green product.
3. I believe I require a lot of time to search for a green product.
4. I believe I am confident about the credibility of the green product label.
5. I believe I can buy a green product for an ecological reason.
**Green product trust (GPT)**
1. I think I feel that green product is generally reliable.
2. I think I feel that green product claims are generally trustworthy.
3. I think I can buy a green product because they are dependable.
4. I think I feel that organic food environmental concern meets my expectation.
5. I think the green organic product keeps promises for environmental protection.
**Green product value (GPV)**
1. I believe green product function provides good value quality.
2. I believe green product environmental performance meets my expectation.
3. I believe I purchase a green product because it has more environmental benefit than the other product.
4. I believe I purchase a green product because it is environmentally friendly.
5. I believe I purchase a green product because it has more environmental concerns than other product.
**Green environmental awareness (GEA)**
1. I think I have a responsibility to protect the environment.
2. I think the environment is getting worse due to pollution.
3. I think the environmental problem will affect human life
4. I think advocating an environmental lifestyle is necessary
5. I think there should be more retail shops selling a green product.
**Green price sensitivity (GPS)**
1. I will prefer to pay higher for the green product that has good quality.
2. The price of green products is in accordance with the benefit.
3. The green product is reasonably priced.
4. I think the prices of green products are in line with the value of the product.
5. I think the prices of green products are more economical
**Green behavioral intention (GBI)**
1. I prefer choosing a product causing lesser pollution.
2. I prefer choosing energy-saving products.
3. I prefer choosing environmentally friendly products.
4. When I have a choice between two equal products, I will prefer the one with less harm to other people.
5. I prefer the environmental protection agency certified products.
6. Over the next month, I will consider buying green products because they are less polluting.

## References

[1] Ogiemwonyi O. (2022). Factors influencing generation Y green behaviour on green products in Nigeria: an application of theory of planned behaviour. Environ. Sustain. Indic..

[2] Watts J. (2019). Human society under urgent threat from loss of Earth's natural life. Guard.

[3] Tanner C., Kaiser F.G., Wöfing Kast S. (2004). Contextual conditions of ecological consumerism: a food-purchasing survey. Environ. Behav..

[4] Wijekoon R., Sabri M.F. (2021). Determinants that influence green product purchase intention and behavior: a literature review and guiding framework. Sustainability.

[5] Zhang N., Xie H. (2015). Toward green IT: modeling sustainable production characteristics for Chinese electronic information industry, 1980–2012. Technol. Forecast. Soc. Change.

[6] Joshi Y., Rahman Z. (2015). Factors affecting green purchase behaviour and future research directions. Int. Strateg. Manag. Rev..

[7] Nekmahmud M. (2020). Tourism Marketing in Bangladesh.

[8] Veleva V., Ellenbecker M. (2001). Indicators of sustainable production: framework and methodology. J. Clean. Prod..

[9] Moisander J. (2007). Motivational complexity of green consumerism. Int. J. Consum. Stud..

[10] Tanner C., Wölfing Kast S. (2003). Promoting sustainable consumption: determinants of green purchases by Swiss consumers. Psychol. Mark..

[11] Barr S., Gilg A. (2006). Sustainable lifestyles: Framing environmental action in and around the home. Geoforum.

[12] Khan M.R., Roy S.K., Pervin M.T. (2022). Retail-based Women entrepreneurship entry model through small business orientation (SBO). JWEE.

[13] Nekmahmud M., Rahman S. (2018). Measuring the competitiveness factors in telecommunication markets. Compet. Emerg. Mark. Mark. Dyn. Age Disruptive Technol..

[14] Chowdhury I., Alamgir M. (2021). Factors influencing green product purchase intention among young consumers in Bangladesh. Soc. Sustain..

[15] Adrita U.W. (2020). Consumers' actual purchase behaviour towards green product: a study on Bangladesh. Int. J. Bus. Innov. Res..

[16] Khan M.R., Roy S.K. (2023). Do primary HR functions model work in emerging economies? Sustainable compact perspective for Bangladeshi RMG industry. Rev. Int. Bus. Strateg..

[17] Roy S.K. (2023). “Green Initiatives and Environmental Sustainability: The Moderating Role of Environmental Values,”.

[18] Dangelico R.M., Vocalelli D. (2017). ‘Green Marketing’: an analysis of definitions, strategy steps, and tools through a systematic review of the literature. J. Clean. Prod..

[19] Sana S.S. (2020). Price competition between green and non green products under corporate social responsible firm. J. Retail. Consum. Serv..

[20] Market S.S.R. (2018).

[21] Khan S.K.R.M.R., Hossain S.M.K. (2016). Determinants of users' satisfaction regarding mobile operators in Bangladesh: an exploratory factor analysis approach on university students. Eur. J. Bus. Manag..

[22] Dabija D.-C., Bejan B.M., Grant D.B. (2018). The impact of consumer green behaviour on green loyalty among retail formats: a Romanian case study. Morav. Geogr. reports.

[23] Roy S.K., Chowdhury S.H., Islam S., Siddique S. (2021).

[24] Magnusson M.K., Arvola A., Hursti U.-K.K., Åberg L., Sjödén P.-O. (2003). Choice of organic foods is related to perceived consequences for human health and to environmentally friendly behaviour. Appetite.

[25] Hassan M.M., Jambulingam M., Alam M.N., Islam M.S. (2019). Redesigning the retention strategy against the emerging turnover of Generation Y: revisiting the long-standing problems from 20Th to 21St century. Int. J. Entrep..

[26] Gurbuz I.B., Nesirov E., Ozkan G. (2021). Investigating environmental awareness of citizens of Azerbaijan: a survey on ecological footprint. Environ. Dev. Sustain..

[27] Cheung M.F.Y., To W.M. (2019). An extended model of value-attitude-behavior to explain Chinese consumers' green purchase behavior. J. Retail. Consum. Serv..

[28] Islam S., Hossain M.S., Roy S.K. (2021).

[29] Kautish P., Paul J., Sharma R. (2019). The moderating influence of environmental consciousness and recycling intentions on green purchase behavior. J. Clean. Prod..

[30] Nagy-Pércsi K., Fogarassy C. (2019). Important influencing and decision factors in organic food purchasing in Hungary. Sustainability.

[31] Fogarassy C., Nagy-Pércsi K., Ajibade S., Gyuricza C., Ymeri P. (2020). Relations between circular economic ‘principles’ and organic food purchasing behavior in Hungary. Agronomy.

[32] Xu X., Hua Y., Wang S., Xu G. (2020). Determinants of consumer's intention to purchase authentic green furniture. Resour. Conserv. Recycl..

[33] Tong Q., Anders S., Zhang J., Zhang L. (2020). The roles of pollution concerns and environmental knowledge in making green food choices: evidence from Chinese consumers. Food Res. Int..

[34] Asif M.H., Zhongfu T., Irfan M., Işık C. (2023). Do environmental knowledge and green trust matter for purchase intention of eco-friendly home appliances? An application of extended theory of planned behavior. Environ. Sci. Pollut. Res..

[35] Sun Y., Li T., Wang S. (2022). ‘I buy green products for my benefits or yours’: understanding consumers' intention to purchase green products. Asia Pacific J. Mark. Logist..

[36] Srivastava V., Gupta A.K. (2023). Price sensitivity, government green interventions, and green product availability triggers intention toward buying green products. Bus. Strateg. Environ..

[37] Yadav R., Pathak G.S. (2017). Determinants of consumers' green purchase behavior in a developing nation: applying and extending the theory of planned behavior. Ecol. Econ..

[38] Khatun A., Roy S.K. (2022). Do green marketing strategies influence green buying intentions? Evidence from developing economy. Int. J. Innov. Sci. Res. Technol..

[39] Hossain A., Khan M.Y.H. (2018).

[40] Qazi W., Qureshi J.A., Raza S.A., Khan K.A., Qureshi M.A. (2020). Impact of personality traits and university green entrepreneurial support on students' green entrepreneurial intentions: the moderating role of environmental values. J. Appl. Res. High Educ..

[41] Ajzen I. (1991). “The theory of planned behaviour. Organizational behaviour and human decision processes.

[42] Hagger M.S. (2019).

[43] Ajzen I., Madden T.J. (1986). Prediction of goal-directed behavior: attitudes, intentions, and perceived behavioral control. J. Exp. Soc. Psychol..

[44] Paul J., Modi A., Patel J. (2016). Predicting green product consumption using theory of planned behavior and reasoned action. J. Retail. Consum. Serv..

[45] Askadilla W.L., Krisjanti M.N. (2017). Understanding Indonesian green consumer behavior on cosmetic products: theory of planned behavior model. Polish J. Manag. Stud..

[46] Liobikienė G., Poškus M.S. (2019). The importance of environmental knowledge for private and public sphere pro-environmental behavior: modifying the value-belief-norm theory. Sustainability.

[47] Oliver H., Volschenk J., Smit E. (2011). Residential consumers in the Cape Peninsula's willingness to pay for premium priced green electricity. Energy Pol..

[48] Han H., Hsu L.-T.J., Sheu C. (2010). Application of the theory of planned behavior to green hotel choice: testing the effect of environmental friendly activities. Tour. Manag..

[49] Lee Y.K. (2017). A comparative study of green purchase intention between Korean and Chinese consumers: the moderating role of collectivism. Sustainability.

[50] Zayed M.F., Gaber H.R., El Essawi N. (2022). Examining the factors that affect consumers' purchase intention of organic food products in a developing country. Sustainability.

[51] Zameer H., Yasmeen H. (2022). Green innovation and environmental awareness driven green purchase intentions. Mark. Intell. Plan..

[52] Román-Augusto J.A., Garrido-Lecca-Vera C., Lodeiros-Zubiria M.L., Mauricio-Andia M. (2022). Green marketing: drivers in the process of buying green products—the role of green satisfaction, green trust, green WOM and green perceived value. Sustainability.

[53] Abeysekera I., Manalang L., David R., Grace Guiao B. (2022). Accounting for environmental awareness on green purchase intention and behaviour: evidence from the Philippines. Sustainability.

[54] Shen M., Wang J. (2022). The impact of pro-environmental awareness components on green consumption behavior: the moderation effect of consumer perceived cost, policy incentives, and face culture. Front. Psychol..

[55] Lavuri R. (2022). Organic green purchasing: moderation of environmental protection emotion and price sensitivity. J. Clean. Prod..

[56] Teixeira S.F., Barbosa B., Cunha H., Oliveira Z. (2022). Exploring the antecedents of organic food purchase intention: an extension of the theory of planned behavior. Sustainability.

[57] Islam M.A., Saidin Z.H., Ayub M.A., Islam M.S. (2022). Modelling behavioural intention to buy apartments in Bangladesh: an extended theory of planned behaviour (TPB). Heliyon.

[58] Ahmed N., Li C., Khan A., Qalati S.A., Naz S., Rana F. (2021). Purchase intention toward organic food among young consumers using theory of planned behavior: role of environmental concerns and environmental awareness. J. Environ. Plan. Manag..

[59] Rustam A., Wang Y., Zameer H. (2020). Environmental awareness, firm sustainability exposure and green consumption behaviors. J. Clean. Prod..

[60] Feil A.A., da Silva Cyrne C.C., Sindelar F.C.W., Barden J.E., Dalmoro M. (2020). Profiles of sustainable food consumption: consumer behavior toward organic food in southern region of Brazil. J. Clean. Prod..

[61] Carfora V. (2019). Explaining consumer purchase behavior for organic milk: including trust and green self-identity within the theory of planned behavior. Food Qual. Prefer..

[62] Qi X., Ploeger A. (2019). Explaining consumers' intentions towards purchasing green food in Qingdao, China: the amendment and extension of the theory of planned behavior. Appetite.

[63] Wang X., Pacho F., Liu J., Kajungiro R. (2019). Factors influencing organic food purchase intention in developing countries and the moderating role of knowledge. Sustainability.

[64] Bashir S., Khwaja M.G., Turi J.A., Toheed H. (2019). Extension of planned behavioral theory to consumer behaviors in green hotel. Heliyon.

[65] Wang H.-J. (2017). Determinants of consumers' purchase behaviour towards green brands. Serv. Ind. J..

[66] Bong Ko S., Jin B. (2017). Predictors of purchase intention toward green apparel products: a cross-cultural investigation in the USA and China. J. Fash. Mark. Manag. An Int. J..

[67] Nguyen T.N., Lobo A., Nguyen B.K. (2018). Young consumers' green purchase behaviour in an emerging market. J. Strateg. Mark..

[68] Anisimova T. (2016). Integrating multiple factors affecting consumer behavior toward organic foods: the role of healthism, hedonism, and trust in consumer purchase intentions of organic foods. J. food Prod. Mark..

[69] Prakash G., Pathak P. (2017). Intention to buy eco-friendly packaged products among young consumers of India: a study on developing nation. J. Clean. Prod..

[70] Wang X., Waris I., Bhutto M.Y., Sun H., Hameed I. (2022). Green initiatives and environmental concern foster environmental sustainability: a study based on the use of reusable drink cups. Int. J. Environ. Res. Public Health.

[71] Sreen N., Purbey S., Sadarangani P. (2018). Impact of culture, behavior and gender on green purchase intention. J. Retail. Consum. Serv..

[72] Barbarossa C., Pastore A. (2015). Why environmentally conscious consumers do not purchase green products: a cognitive mapping approach. Qual. Mark. Res. Int. J..

[73] Ajzen I. (2015). The theory of planned behaviour is alive and well, and not ready to retire: a commentary on Sniehotta, Presseau, and Araújo-Soares. Health Psychol. Rev..

[74] Ajzen I. (1985).

[75] Arvola A. (2008). Predicting intentions to purchase organic food: the role of affective and moral attitudes in the Theory of Planned Behaviour. Appetite.

[76] Jain V.K., Arya V., Sharma P. (2021). Social media and sustainable behavior: a decision making framework using interpretive structural modeling (ISM). J. Content Community Commun..

[77] Bondos I. (2016). Store price image–the power of perception. Int. J. Synerg. Res..

[78] Hamzaoui Essoussi L., Linton J.D. (2010). New or recycled products: how much are consumers willing to pay?. J. Consum. Mark..

[79] Lim W.M., Yong J.L.S., Suryadi K. (2014). Consumers' perceived value and willingness to purchase organic food. J. Glob. Mark..

[80] Sharaf M.A., Perumal S. (2018). How does green products? Price and availability impact Malaysians? Green purchasing behavior?. J. Soc. Sci. Res..

[81] Biswas A., Roy M. (2015). Green products: an exploratory study on the consumer behaviour in emerging economies of the East. J. Clean. Prod..

[82] Lin P.-C., Huang Y.-H. (2012). The influence factors on choice behavior regarding green products based on the theory of consumption values. J. Clean. Prod..

[83] Karatu V.M.H., Mat N.K.N. (2015). The mediating effects of green trust and perceived behavioral control on the direct determinants of intention to purchase green products in Nigeria. Mediterr. J. Soc. Sci..

[84] Zhuang W., Luo X., Riaz M.U. (2021). On the factors influencing green purchase intention: a meta-analysis approach. Front. Psychol..

[85] Chen Y.-S., Lin C.-Y., Weng C.-S. (2015). The influence of environmental friendliness on green trust: the mediation effects of green satisfaction and green perceived quality. Sustainability.

[86] Kim D.J., Ferrin D.L., Rao H.R. (2008). A trust-based consumer decision-making model in electronic commerce: the role of trust, perceived risk, and their antecedents. Decis. Support Syst..

[87] Karatu V.M.H., Nik Mat N.K. (2015).

[88] Alshura M.S., Zabadi A.M. (2016). Impact of green brand trust, green brand awareness, green brand image, and green perceived value on consumer's intension to use green products: an empirical study of jordanian consumers. Int. J. Adv. Res..

[89] Dong X., Zhao H., Li T. (2022). The role of live-streaming e-commerce on consumers' purchasing intention regarding green agricultural products. Sustainability.

[90] Khor K.S., Udin Z.M., Ramayah T., Hazen B.T. (2016). Reverse logistics in Malaysia: the contingent role of institutional pressure. Int. J. Prod. Econ..

[91] Patterson P.G., Spreng R.A. (1997). Modelling the relationship between perceived value, satisfaction and repurchase intentions in a business‐to‐business, services context: an empirical examination. Int. J. Serv. Ind. Manag..

[92] Yaacob M.R., Zakaria A. (2011). Customers' awareness, perception and future prospects of green products in Pahang, Malaysia. J. Commer..

[93] Chen Y., Chang C. (2013).

[94] Kong W., Harun A., Sulong R.S., Lily J. (2014). The influence of consumers perception of green products on green purchase intention. Int. J. Asian Soc. Sci..

[95] Ogiemwonyi O. (2020). Green product as a means of expressing green behaviour: a cross-cultural empirical evidence from Malaysia and Nigeria. Environ. Technol. Innov..

[96] Bhaskaran S., Polonsky M., Cary J., Fernandez S. (2006). Environmentally sustainable food production and marketing: opportunity or hype?. Br. Food J..

[97] Chu K.M. (2018). “Mediating influences of attitude on internal and external factors influencing consumers' intention to purchase organic foods in China. Sustainability.

[98] Khaola P.P., Potiane B., Mokhethi M. (2014). Environmental concern, attitude towards green products and green purchase intentions of consumers in Lesotho. Ethiop. J. Environ. Stud. Manag..

[99] Kinnear T.C., Taylor J.R., Ahmed S.A. (1974). “Ecologically concerned consumers: who are they? Ecologically concerned consumers can be identified,”. J. Mark..

[100] Tan Y., Zhu Z. (2022). The effect of ESG rating events on corporate green innovation in China: the mediating role of financial constraints and managers' environmental awareness. Technol. Soc..

[101] Darvishmotevali M., Altinay L. (2022). Green HRM, environmental awareness and green behaviors: the moderating role of servant leadership. Tour. Manag..

[102] Synodinos N.E. (1990). Environmental attitudes and knowledge: a comparison of marketing and business students with other groups. J. Bus. Res..

[103] Dunlap R.E., Van Liere K.D. (1978). The ‘new environmental paradigm. J. Environ. Educ..

[104] Cordano M., Welcomer S.A., Scherer R.F. (2003). An analysis of the predictive validity of the new ecological paradigm scale. J. Environ. Educ..

[105] Yi S. (2019). Determinants of consumers’ purchasing behavior for certified aquaculture products in South Korea. Sustainability.

[106] Zorić J., Hrovatin N. (2012). Household willingness to pay for green electricity in Slovenia. Energy Pol..

[107] Chen C.-C., Chen C.-W., Tung Y.-C. (2018). Exploring the consumer behavior of intention to purchase green products in belt and road countries: an empirical analysis. Sustainability.

[108] Ham M., Mrčela D., Horvat M. (2016). Insights for measuring environmental awareness. Ekon. Vjesn. Rev. Contemp. Entrep. Business, Econ. Issues.

[109] Aman Z., Rahim A.R.A., Din A.K. (2015). Generation Y perceptions of employment in the plantation sector. Int. J. Recent Adv. Organ. Behav. Decis. Sci..

[110] Hedlund T. (2011). The impact of values, environmental concern, and willingness to accept economic sacrifices to protect the environment on tourists' intentions to buy ecologically sustainable tourism alternatives. Tour. Hosp. Res..

[111] Roy S.K., Ahmed J. (2016). A relational study of communication, reputation and cooperation on relationship satisfaction in the context of apparel sector in Bangladesh. Br. Open J. Bus. Adm..

[112] Khan M.R., Roy S.K., Hossain S.M.K. (2019). Factors affecting garments employees perception on job performance: evidence from Bangladesh. Int. J. Manag. Sustain..

[113] Chowdhury S.H., Roy S.K., Arafin M., Siddiquee S. (2019). Green HR practices and its impact on employee work satisfaction-A case study on IBBL, Bangladesh. Int. J. Res. Innov. Soc. Sci. Delhi.

[114] Faul F., Erdfelder E., Buchner A., Lang A.G. (2009). Statistical power analyses usingG* Power 3.1: tests for correlation and regression analyses. Behav. Res..

[115] Hulland J., Baumgartner H., Smith K.M. (2018). Marketing survey research bestpractices: evidence and recommendations from a review of JAMS articles. J. ofthe Acad. Mark. Sci..

[116] Tonglet M., Phillips P.S., Read A.D. (2004). Using the Theory of Planned Behaviour to investigate the determinants of recycling behaviour: a case study from Brixworth, UK. Resour. Conserv. Recycl..

[117] Soomro B.A., Ghumro I.A., Shah N. (2020). Green entrepreneurship inclination among the younger generation: an avenue towards a green economy. Sustain. Dev..

[118] Thien L.M. (2020). Assessing a second-order quality of school life construct using partialleast squares structural equation modelling approach. Int. J. ofResearch Method Educ..

[119] Sarstedt M., Hair J.F., Cheah J.H., Becker J.M., Ringle C.M. (2019). How tospecify, estimate, and validate higher-order constructs in PLS-SEM. Australas. J..

[120] Jarvis C., Scott B., Machenzie A. (2003). Critical review of construct indicators andmeasurement model misspecification in marketing and consumer research. Journalof Consum. Res..

[121] Roy S.K. (2023).

[122] Chin W., Cheah J.-H., Liu Y., Ting H., Lim X.-J., Cham T.H. (2020). Demystifying the role of causal-predictive modeling using partial least squares structural equation modeling in information systems research. Ind. Manag. Data Syst..

[123] Becker J.M., Ringle C.M., Sarstedt M. (2018). Estimating moderating effects in PLSSEM and PLSc-SEM: interaction term generation* data treatment. J. AppliedStructural Equ. Model..

[124] Hair J.F., Risher J.J., Sarstedt M., Ringle C.M. (2019). When to use and how toreport the results of PLS-SEM. Eur. Bus. Rev..

[125] Roy S.K. (2023).

[126] Roy S.K. (2023). E-learning Portal Success in higher education organizations: a multi-group comparison. J. Soc. Humanit. Educ..

[127] Spector P.E. (2006). Method variance in organizational research: truth or urbanlegend?. Organ. Res. Methods.

[128] Kline R.B. (2015).

[129] MacKenzie S.B., Podsakoff P.M. (2012). Common method bias in marketing: causes,mechanisms, and procedural remedies. J. Retail..

[130] Kock N. (2015). Common method bias in PLS-SEM: a full collinearity assessmentapproach. Int. J. e-Collaboration.

[131] Hair J.F., Hult G.T.M., Ringle C., Sarstedt M. (2017).

[132] Fornell C., Larcker D.F. (1981). Evaluating structural equation models withunobservable variables and measurement error. J. Mark. Res..

[133] Becker J.M., Klein K., Wetzels M. (2012). Hierarchical latent variable models in PLSSEM: guidelines for using reflective-formative typemodels. Long Range Plann.

[134] Roy S.K. (2023). Impact of SMS advertising on purchase intention for young consumers. Int. J. Financ. Accounting, Manag..

[135] Roy S.K., Islam S. (2023). Influence of confidence factors on e-learning acceptance for future use by university students: evidence from an emerging economy. SN Soc. Sci..

[136] Roy S.K. (2022). The impact of age, gender, and ethnic diversity on organizational performance: an empirical study of Bangladesh's banking sector. Int. J. Financ. Accounting, Manag..

[137] Henseler J., Ringle C.M., Sarstedt M. (2015). A new criterion for assessingdiscriminant validity in variance-based structural equation modeling. J. theAcademy Mark. Sci..

[138] Dul J. (2016). http://cran.r-project.

[139] Roy S.K., Khan M.R., Shanto N.I. (2023).

[140] Anvar M., Venter M. (2014). Attitudes and purchase behaviour of green products among generation Y consumers in South Africa. Mediterr. J. Soc. Sci..

[141] Ariffin S., Yusof J.M., Putit L., Shah M.I.A. (2016). Factors influencing perceived quality and repurchase intention towards green products. Procedia Econ. Financ..

[142] Chou C.-J. (2014). Hotels' environmental policies and employee personal environmental beliefs: interactions and outcomes. Tour. Manag..

[143] Malik M.I. (2019). Contradictory results on environmental concern while re-visiting green purchase awareness and behavior. Asia Pacific J. Innov. Entrep..

[144] Chowdhury S., Roy S. (2015). Evaluating the impact of insurance companies in the development of insurance practices in Bangladesh. Sch. J. Bus. Soc. Sci..

[145] Peano C., Baudino C., Tecco N., Girgenti V. (2015). Green marketing tools for fruit growers associated groups: application of the Life Cycle Assessment (LCA) for strawberries and berry fruits ecobranding in northern Italy. J. Clean. Prod..

[146] Chase D., Smith T.K. (1992). Consumers keen on green but marketers don't deliver. Advert. Age.

[147] Chkanikova O., Lehner M. (2015). Private eco-brands and green market development: towards new forms of sustainability governance in the food retailing. J. Clean. Prod..

