# Supplementary material for: Impact of green factors on undergraduate students’ green behavioral intentions: A hybrid two-stage modeling approach
Source: Heliyon. 2023 Oct 5;9(10):e20630. doi: 10.1016/j.heliyon.2023.e20630 (PMC10582314; doi:10.1016/j.heliyon.2023.e20630)
Supplement: Multimedia component 1 [file mmc1.docx]

**Green factors and green behavioral intentions: A hybrid two-stage modeling approach**

Figure 01 represents the proposed research model.

| Green Personal Attitude (GPA)  Green TPB Perception (TPB)  Green Behavioral Intention (GBI)  Green Behavioral Control (GBC)  Green Subjective Norms (GSN)  Green Product Trust (GPT)  Green Environmental Awareness (GEA)  Green Price Sensitivity (GPS)  Green Product Value (GPV) |
| --- |
|  |
| Figure 01: Conceptual framework |

Table 01

Table 01: Undergraduates demographics

| Variables | Categories | Frequency | Percent |
| --- | --- | --- | --- |
| Gender | Male | 221 | 57.90 |
|  | Female | 161 | 42.10 |
| Academic year | 1st year | 35 | 9.20 |
|  | 2nd year | 102 | 26.70 |
|  | 3rd year | 199 | 52.10 |
|  | 4th year | 46 | 12.00 |

Table 02

Table 02: Assessment of construct validity

| Constructs | Items | λ | α | CR | AVE |
| --- | --- | --- | --- | --- | --- |
| Green Personal Attitude (GPA) | GPA1 | 0.868 | 0.919 | 0.937 | 0.712 |
|  | GPA2 | 0.844 |  |  |  |
|  | GPA3 | 0.851 |  |  |  |
|  | GPA4 | 0.833 |  |  |  |
|  | GPA5 | 0.813 |  |  |  |
|  | GPA6 | 0.853 |  |  |  |
| Green Behavioral Control (GBC) | GBC1 | 0.828 | 0.926 | 0.944 | 0.771 |
|  | GBC2 | 0.920 |  |  |  |
|  | GBC3 | 0.894 |  |  |  |
|  | GBC4 | 0.866 |  |  |  |
|  | GBC5 | 0.881 |  |  |  |
| Green Subjective Norms (GSN) | GSN1 | 0.855 | 0.918 | 0.939 | 0.754 |
|  | GSN2 | 0.888 |  |  |  |
|  | GSN3 | 0.873 |  |  |  |
|  | GSN4 | 0.857 |  |  |  |
|  | GSN5 | 0.868 |  |  |  |
| Green Price Sensitivity (GPS) | GPS1 | 0.852 | 0.915 | 0.937 | 0.747 |
|  | GPS2 | 0.888 |  |  |  |
|  | GPS3 | 0.872 |  |  |  |
|  | GPS4 | 0.845 |  |  |  |
|  | GPS5 | 0.864 |  |  |  |
| Green Product Trust (GPT) | GPT1 | 0.869 | 0.918 | 0.938 | 0.753 |
|  | GPT2 | 0.863 |  |  |  |
|  | GPT3 | 0.870 |  |  |  |
|  | GPT4 | 0.871 |  |  |  |
|  | GPT5 | 0.866 |  |  |  |
| Green Product Value (GPV) | GPV1 | 0.894 | 0.932 | 0.948 | 0.785 |
|  | GPV2 | 0.906 |  |  |  |
|  | GPV3 | 0.869 |  |  |  |
|  | GPV4 | 0.883 |  |  |  |
|  | GPV5 | 0.878 |  |  |  |
| Green Environmental Awareness (GEA) | GEA1 | 0.882 | 0.926 | 0.944 | 0.771 |
|  | GEA2 | 0.894 |  |  |  |
|  | GEA3 | 0.862 |  |  |  |
|  | GEA4 | 0.866 |  |  |  |
|  | GEA5 | 0.887 |  |  |  |
| Green Behavioral Intention (GBI) | GBI1 | 0.919 | 0.956 | 0.965 | 0.820 |
|  | GBI2 | 0.914 |  |  |  |
|  | GBI3 | 0.913 |  |  |  |
|  | GBI4 | 0.914 |  |  |  |
|  | GBI5 | 0.873 |  |  |  |
|  | GBI6 | 0.898 |  |  |  |

Table 03

Table 03: Assessment of discriminant.

| Fornell-Larcker Criterion | | | | | | | | |
| --- | --- | --- | --- | --- | --- | --- | --- | --- |
|  | GBC | GBI | GEA | GPA | GPS | GPT | GPV | GSN |
| GBC | **0.878** |  |  |  |  |  |  |  |
| GBI | 0.735 | **0.905** |  |  |  |  |  |  |
| GEA | 0.672 | 0.786 | **0.878** |  |  |  |  |  |
| GPA | 0.613 | 0.774 | 0.682 | **0.844** |  |  |  |  |
| GPS | 0.628 | 0.793 | 0.682 | 0.670 | **0.864** |  |  |  |
| GPT | 0.596 | 0.775 | 0.693 | 0.750 | 0.636 | **0.868** |  |  |
| GPV | 0.633 | 0.789 | 0.691 | 0.736 | 0.684 | 0.715 | **0.886** |  |
| GSN | 0.639 | 0.796 | 0.701 | 0.671 | 0.710 | 0.738 | 0.715 | **0.868** |
| HTMT Ratio | | | | | | | | |
|  | GBC | GBI | GEA | GPA | GPS | GPT | GPV | GSN |
| GBC |  |  |  |  |  |  |  |  |
| GBI | 0.782 |  |  |  |  |  |  |  |
| GEA | 0.726 | 0.834 |  |  |  |  |  |  |
| GPA | 0.665 | 0.825 | 0.738 |  |  |  |  |  |
| GPS | 0.681 | 0.847 | 0.741 | 0.728 |  |  |  |  |
| GPT | 0.645 | 0.826 | 0.750 | 0.815 | 0.692 |  |  |  |
| GPV | 0.681 | 0.836 | 0.743 | 0.794 | 0.741 | 0.771 |  |  |
| GSN | 0.692 | 0.849 | 0.759 | 0.728 | 0.773 | 0.804 | 0.771 |  |
| Note: The above matrix's diagonal values (bold) represent the square roots of AVEs, whereas the off-diagonal values represent correlations between the latent components. | | | | | | | | |

Table 04

Table 04: HOC assessment

| HOC | LOCs | VIF | OW | t-values | 95% BCa-CIs |
| --- | --- | --- | --- | --- | --- |
| Personality traits (PT) | GPA | 2.302 | 0.406 | 6.741* | [0.324, 0.520] |
|  | GSN | 2.155 | 0.452 | 8.883* | [0.336, 0.554] |
|  | GBC | 2.007 | 0.272 | 5.303* | [0.181, 0.371] |
| Note: OW = Outer weight, LOC = Lower order construct, HOC = Higher order construct, *p<0.001. | | | | | |

Figure 02

| 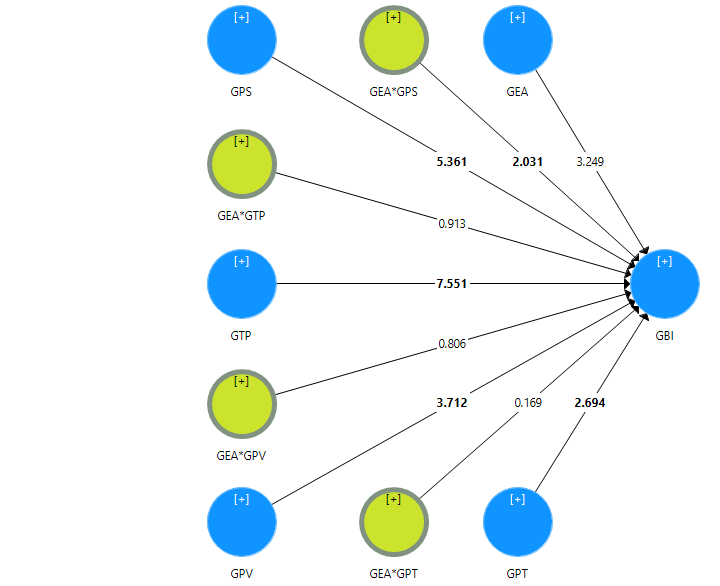 |
| --- |
|  |
| Figure 02: Results of the structural model |

Table 05

Table 05: Assessment of hypotheses relationships

| Hypotheses |  | β | t-values | p-values | Supported |
| --- | --- | --- | --- | --- | --- |
|  | *Direct effects* |  |  |  |  |
| H1 | GTP -> GBI | 0.420 | 7.551 | 0.000 | Yes |
| H2 | GPS -> GBI | 0.204 | 5.361 | 0.000 | Yes |
| H3 | GPT -> GBI | 0.122 | 2.694 | 0.008 | Yes |
| H4 | GPV -> GBI | 0.137 | 3.712 | 0.000 | Yes |
|  | *Moderation effects* |  |  |  |  |
| H5 | GEA*GTP -> GBI | -0.045 | 0.913 | 0.363 | No |
| H6 | GEA*GPS -> GBI | 0.099 | 2.031 | 0.045 | Yes |
| H7 | GEA*GPT -> GBI | 0.009 | 0.169 | 0.867 | No |
| H8 | GEA*GPV -> GBI | -0.036 | 0.806 | 0.422 | No |

Figure 03

|  |
| --- |
|  |
| Figure 03: Moderation effects of GEA |

Table 6

Table 06: Bottleneck table

|  | **CE-FDH** | | | | **CR-FDH** | | | |
| --- | --- | --- | --- | --- | --- | --- | --- | --- |
| **GBI** | **GTP** | **GPS** | **GPT** | **GPV** | **GTP** | **GPS** | **GPT** | **GPV** |
| 0 | NN | NN | NN | NN | 3.0 | NN | 0.3 | NN |
| 10 | 14.3 | 9.8 | 13.4 | 3.4 | 10.1 | 7.4 | 6.2 | 2.5 |
| 20 | 27.3 | 30.0 | 16.7 | 24.9 | 17.3 | 14.9 | 12.1 | 9.5 |
| 30 | 27.3 | 30.0 | 16.7 | 24.9 | 24.5 | 22.5 | 18.0 | 16.4 |
| 40 | 48.2 | 50.0 | 33.3 | 28.6 | 31.7 | 30. | 23.9 | 23.4 |
| 50 | 48.2 | 50.0 | 33.3 | 28.6 | 38.9 | 37.5 | 29.8 | 30.4 |
| 60 | 48.2 | 50.0 | 33.3 | 39.4 | 46.4 | 45.1 | 35.7 | 37.3 |
| 70 | 57.7 | 50.0 | 46.7 | 46.4 | 53.2 | 52.3 | 41.6 | 44.3 |
| 80 | 60.4 | 50.0 | 54.0 | 46.4 | 60.4 | 60.1 | 47.5 | 51.2 |
| 90 | 66.0 | 66.7 | 54.0 | 64.3 | 67.6 | 67.7 | 53.4 | 58.2 |
| 100 | 71.4 | 76.9 | 54.0 | 64.3 | 74.8 | 75.2 | 59.3 | 65.1 |
| *effect size, d* | 0.435 | 0.432 | 0.331 | 0.342 | 0.389 | 0.375 | 0.298 | 0.305 |
| *p-value* | 0.000 | 0.000 | 0.000 | 0.000 | 0.000 | 0.000 | 0.000 | 0.000 |
| *c-accuracy* | 100% | 100% | 100% | 100% | 96.10% | 94.00% | 98.20% | 97.40% |

Figure 04

| 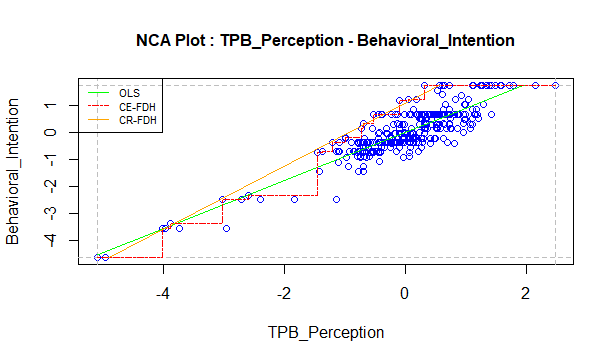 | 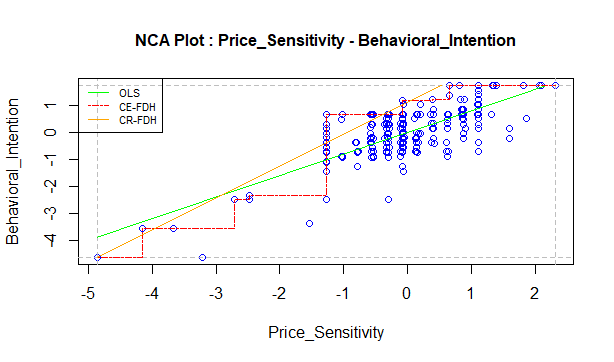 |
| --- | --- |
| 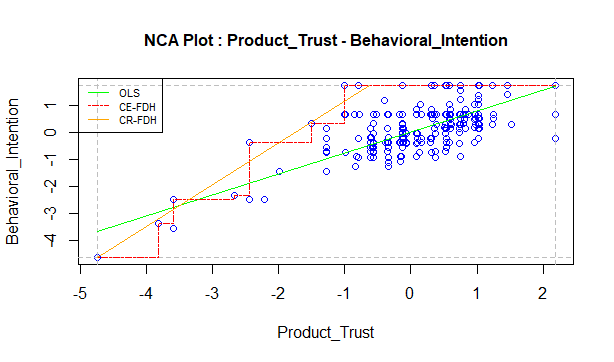 | 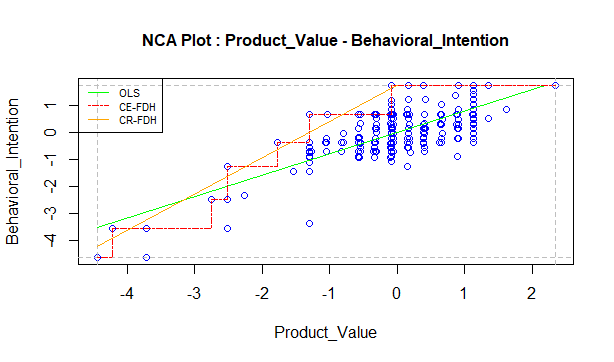 |
|  |  |
| Figure 04: GTP, GPS, GPT, GPV, and GBI; OLS regression line; Scatter plot, CR-FDH ceiling line | |
